# Peace and Local Saturday Societies. The New Value of Money

**Published:** 1919-07-26

**Authors:** 


### LEICESTER ROYAL INFIRMARY.

### Peace and Local Saturday Societies. The New Value of Money.

At the half-yearly meeting of the Leicester and County Saturday Hospital Society, held at the Royal Infirmary on Peace Day, two hundred delegates resolved, without a dissentient, to recommend the contributors to double the existing weekly contributions. In the words of the Committee, this change was necessitated by the higher cost of maintenance of the Leicester Royal Infirmary and the Society's Convalescent Homes, as well as by the decision of the Committee to provide convalescent home treatment for members' children—a decision which would entail further large additional charges upon the Society's income, but would be of great advantage.

Mr. J. Gipson Clarke, J.P., the President, in submitting the recommendation, stated that it would be well understood that the expenditure of the infirmary had rapidly risen, while the Society's Convalescent Home work was at present conducted with a deficit. The large attendance showed the interest of the members and convinced him that they had not made a mistake in fixing their meeting for Peace Day. They could in no better way celebrate the Great Peace than by bringing their contributions to the new standard value of money, and by extending their work so as to include the convalescent care and treatment of the rising generation. The recommendation was received with approval from every section of the contributors.

The meeting was notable by the fact that employers as well as employed were in complete agreement on the proposal. An employer of 800 hands in a town which was even then raising funds for the establishment of a cottage hospital stated that on the receipt of the notice of meeting he convened a general meeting of the employees, and in ten minutes a resolution to double the weekly contributions was passed without a dissentient. If thirteen miles away from the centre they were unanimous in the reasonableness of the proposal, he could not doubt that in the City Headquarters they would be as completely unanimous. Replying to an employer in another distant town with a local hospital, the Chairman stated that under the existing arrangement with that hospital for an equal share of the contributions, the local hospital would at once receive a considerable accession of income without expenditure or effort, as the parent society would organise the new system on the same lines as the old. The resolution was unanimously adopted.

The half-yearly report of the Society was submitted. During the four and a-half years in which the Desford Home for Men had been placed at the disposal of the War Office as an auxiliary hospital, over 1,900 sick and wounded soldiers had been received. The Government grants for their treatment on the agreed scale were £11,893, the actual cost to the Society was £14,773, which showed a charge of £2,880 upon the Society's income. The Home was now in course of redecoration and refurnishment, at a cost of £500, and the War Office were being appealed to for a financial grant. The Home would shortly be reopened for fifty-six men to commence with, and the formal unveiling of the plaque to the memory of Sir Edward Wood, the Society's founder and first President, would take place on the opening day. Two hundred female and 137 male members had received convalescent treatment at the Swithland Home, and 114 members had been sent to other homes during the six months—451 patients in all. One hundred and one military patients had also been received, making a grand total of 552. The half-year's expenditure was £3,632.

Since the last report, twenty-one firms employing 533 hands had been added to membership. With the conclusion of Peace the Society's work would call for further developments, and would be the more needed. The report was adopted, and, on the invitation of the President, the delegates enjoyed tea provided through his hospitality.

